# Clinical features of children with enthesitis-related juvenile idiopathic arthritis / juvenile spondyloarthritis followed in a French tertiary care pediatric rheumatology centre

**DOI:** 10.1186/s12969-018-0238-9

**Published:** 2018-04-02

**Authors:** Maxime Goirand, Sylvain Breton, Frédéric Chevallier, Ngoc-Phoi Duong, Florence Uettwiller, Isabelle Melki, Richard Mouy, Carine Wouters, Brigitte Bader-Meunier, Chantal Job-Deslandre, Pierre Quartier

**Affiliations:** 1 Pediatric Immunology, Hematology, and Rheumatology Unit, Centre de Référence pour les Rhumatismes Inflammatoires et les Maladies Auto-Immunes Systémique Rare de l’Enfant (RAISE) ; Necker-Enfants Malades Hospital, Assistance Publique Hôpitaux de Paris, 149, rue de Sèvres, 75743 Cedex15 Paris, France; 2 Paris Descartes University, 12 rue de l’Ecole de Médicine, 75006 Paris, France; 3 Pediatric Radiology Department, Necker-Enfants Malades Hospital, Assistance Publique Hôpitaux de Paris, 149, rue de Sèvres, 75743 Cedex 15 Paris, France; 4 UFR SMBH Paris 13, 74 rue Marcel Cachin, 93017 Cedex Bobigny, France; 5 Service de Réanimation Néonatale, Centre Hospitalier Intercommunal de Créteil, 40 avenue de Verdun, 94000 Créteil, France; 6 Imagine Institute, 24 boulevard du Montparnasse, 75015 Paris, France; 7 General Pediatrics, Infectious Disease, and Internal Medicine Unit, Robert Debré Hospital, Assistance Publique Hôpitaux de Paris, 48 boulevard Sérurier, 75019 Paris, France; 8 INSERM UMR 1163, Laboratory of Neurogenetics and Neuroinflammation, Paris, France; 9 GOIRAND, CETD et EMASP pédiatrique, Hôpital Robert Debré, 48, Boulevard Serrurier, 75019 Paris, France

**Keywords:** Juvenile spondyloarthritis, Enthesitis related arthritis, Juvenile idiopathic arthritis, Anti-TNF treatment, Classification criteria, Prognostic factor

## Abstract

**Background:**

Childhood-onset spondyloarthropathies usually start with enthesitis and peripheral arthritis. However, axial disease may develop afterward. Patients are most often classified, following revised (Edmonton 2011) ILAR criteria, as enthesitis-related arthritis, psoriatic arthritis, or unclassified juvenile idiopathic arthritis, particularly in cases of psoriasis in the patient or a first-degree relative. In adults, peripheral spondyloarthritis is classified by ASAS criteria.

**Methods:**

We retrospectively studied patients with childhood-onset spondyloarthropathies followed for more than one year in our referral centre. We did not exclude patients with a personal or familial history of psoriasis.

**Results:**

We included 114 patients followed between January 2008 and December 2015 for a median of 2.5 years (IQR = 2.3). Sixty-nine per-cent of patients fulfilled the revised ILAR classification criteria for enthesitis-related arthritis, and 92% the ASAS criteria for peripheral spondyolarthritis (*p* <  0.001). Axial disease and sacroiliitis were rare at disease onset. However, they appeared during follow-up in 63% and 47% of cases respectively, after a median disease duration of 2.6 (IC 95% [2.2–4.4]) and 5.3 years (IC 95% [4.1–7.7]), respectively. Multivariable analysis showed that familial history of spondyloarthritis was associated with the presence of sacroiliitis and active disease at the latest follow-up (OR = 3.61 [1.5–8.7], *p* <  0.01 and 2.98 [1.2–7.3], *p* = 0.02, respectively).

**Conclusion:**

Axial involvement developed in most patients within five years. Revised Edmonton criteria were less sensitive than ASAS criteria to classify patients as having childhood-onset spondyloarthropathies. The main risk factor for both sacroiliitis and persistent active disease was a familial history of spondyloarthritis.

**Electronic supplementary material:**

The online version of this article (10.1186/s12969-018-0238-9) contains supplementary material, which is available to authorized users.

## Background

Juvenile idiopathic arthritis (JIA) is a heterogeneous group of inflammatory joint diseases of unknown aetiology, defined by the presence of chronic arthritis before the age of 16 years. It is the most common disease treated in paediatric rheumatology clinics with a prevalence of 16 to 150/100,000 children [1]. Each form of JIA is defined by specific and non specific clinical characteristics [2]. The last published classification criteria for JIA are the 2002 Edmonton criteria [2]. Enthesitis-related arthritis (ERA), one JIA subcategory, and psoriatic arthritis (PsA) comprise most cases of paediatric spondyloarthritis (SpA) (Additional file 1: Table S1). ERA represents up to 20% of JIA cases and usually starts after six years of age [1, 3]. The principle of the Edmonton classification is that all categories of JIA are mutually exclusive, which is reflected by the presence of the undefined juvenile arthritis category. Patients with juvenile SpA (JSpA), who do not fulfil either the ERA or PsA criteria are classified in this last category [2].

SpA is a heterogeneous group of inflammatory diseases, first identified in adults. It is characterised by a strong association with HLA-B27 [4]. The disease spectrum is large, ranging from reactive arthritis to ankylosing spondylitis (AS). SpA is defined by a high risk of axial disease, typically with sacroiliitis. Some patients present extra-articular disease, such as uveitis or psoriasis. The paediatric form differs from that of adults by a more peripheral pattern of arthritis with less frequent axial disease, at least at disease onset. Evolution to ankylosis is reported in one third of cases after several years of disease [5]. The reported frequency of ankylosis varies greatly, depending on the study, in 9% to 75% of cases [6]. Little is known about the long-term outcome of JSpA. The remission rate is reported to be between 17% and 37%, with a severe handicap in 4% to 52% of cases [6]. Few studies have compared adult to juvenile SpA. One study showed a worse outcome with more disability for JSpA [7].

In adults, the first set of criteria identified only late stage axial SpA, with defined X-ray sacroiliitis (New York criteria) [4]. Various criteria have been progressively proposed to classify the disease at an earlier stage. Until recently, most criteria mainly focused on axial disease with little interest in peripheral disease. Recently the Assessment of Spondylarthritis International Society (ASAS) published two classification criteria sets, one for axial forms in 2009 [8] and one for peripheral forms in 2011 [9]. Peripheral criteria are: the presence of arthritis, dactylitis, or enthesitis, associated with at least one major criterion (uveitis, psoriasis, inflammatory bowel disease, infectious trigger, presence of HLA-B27, or sacroiliitis) or at least two minor criteria (arthritis, enthesitis, dactylitis, inflammatory back pain, or a familial history of SpA) [9]. In children, the situation is more complex, with some studies using adult and others paediatric criteria, mainly the revised Edmonton criteria for JIA. However paediatric cases of SpA do not always fulfil the Edmonton criteria of ERA: some are classified as PsA or undifferentiated JIA. The lack of diagnostic consensus, with multiple classification criteria, is a limiting factor for meta-analyses [4, 10].

We performed a retrospective study of all JSpA patients followed in a paediatric rheumatology tertiary care centre at Necker Hospital in Paris to determine the initial presentation and outcome of patients, including response to treatment, and analyse the risk factors for axial disease or active disease at the last follow up. In addition, we analysed the proportion of patients diagnosed as ERA or SpA according to the revised Edmonton classification criteria and the latest ASAS criteria, respectively.

## Methods

### Patients

In this retrospective study, we enrolled all patients evaluated at a French national reference centre for juvenile arthritis in Necker-Enfants Malades hospital and the paediatric rheumatology outpatient clinic of Saint-Vincent-de-Paul Hospital between January 2008 and December 2015. Patients were recruited through the “Centre de Référence des Maladies Rares (CEMARA)” database project [11]. The recording of patient, parent information, data collection and analysis were conducted in accordance with French national guidelines. CEMARA has been authorized by the French National Committee on Informatics and Liberty. According to French legislation, patients and parents were informed, but informed consent was not required for a retrospective analysis.

The inclusion criteria were having ERA or JSpA, with disease onset at an age of less than 16 years, diagnosed by the attending physician, and confirmed at the latest follow-up visit. We did not exclude patients with a personal or familial history of psoriasis. Patients followed for less than one-year or primarily diagnosed with inflammatory bowel disease were excluded.

### Data collection

For each patient, we collected information concerning gender, age at disease onset, age at first visit, personal and familial history of inflammatory and auto-immune disease and psoriasis, presence of the HLA-B27 allele, description of the clinical course, date of first axial involvement, and biological and radiological tests from the medical chart. Data collection was performed two times by one investigator, first from the computerized medical chart and then from the paper chart. We also analysed the response to various treatments and adverse events. We noted any history of uveitis, psoriasis, or IBD to highlight extra-articular manifestation. Imaging data were recorded and analysed as described below.

### Clinical and radiological assessment

We defined axial disease by the presence of at least one criterion among: 1) inflammatory low-back pain or inflammatory dorsal pain lasting more than one month; 2) limited spine mobility, defined by a Schober index < 10 + 4 cm; 3) sacroiliac pain at examination or alternating buttock pain; or 4) presence of axial disease by any available radiological examination. Active sacroiliitis was defined as sacroiliac pain at examination, alternating buttock pain or the presence of an active lesion by MRI. A joint was considered to be active if at least two criteria among inflammatory pain, limited mobility, and/or swelling were present. Enthesitis was defined as chronic inflammatory pain or tenderness at the site of principal tendon insertion: plantar fascia insertion at the metatarsal heads and calcaneum, Achilles tendon insertion at the calcaneum, quadriceps insertion at the patella and proximal tibia, pelvis (anterior superior iliac spine, iliac crest), and greater trochanter.

An experienced pediatric radiologist (SB) reviewed all available sacroiliac MRI, assessing the presence of acute lesions (synovitis or articular effusion, bone oedema, or enthesitis) and chronic lesions (bone erosion, bone sclerosis, degenerative fatty infiltration of the bone, or ankylosis). Active sacroiliitis by MRI was defined by the presence of at least one acute lesion. Our expert radiologist did not systematically review other radiological examinations, such as CT-scans scan or standard radiography. Interrater reliability of MRI evaluations was measured with kappa coefficients. A result of k <  0.40 indicated poor agreement, 0.40–0.59 fair agreement, 0.60–0.74 moderate agreement, and 0.75–1.00 excellent agreement [12].

### Assessment of response to treatment

Clinical response to treatment was evaluated based on the information recorded from the medical chart: reduction of arthritis and clinical symptoms for at least six months, when available, and the treating physician evaluation. Inactive disease was defined as the absence of arthritis and enthesitis during clinical examination. Clinical remission was defined as the absence of symptoms, noted by the treating rheumatologist, for at least six months, with or without treatment.

### Prognostic factors

We analysed the following variables; masculine gender, presence of HLA-B27, age of first symptoms under 12 years, hip arthritis, presence of enthesitis, and family history of SpA.

#### Statistical analysis

All statistical analyses were performed with R (version 3.2.2). Comparisons between patient groups were performed using the chi-squared test and Fisher’s exact test for qualitative variables, and Student’s t-test for quantitative variables. Survival analyses were performed using the Kaplan Meier method. Multivariable analysis, using a logistic model, was performed to determine variables, including disease severity indicators. The predictors used in the final model were those showing a significant correlation (*p* ≤ 0.05) with sacroiliac (SI) involvement in the univariate analysis.

## Results

### Baseline characteristics

**Fig. 1:**
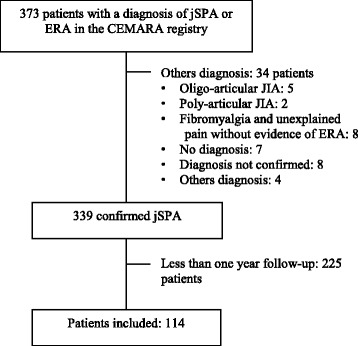
Flow chart. CEMARA, a French information system for rare disease. ERA, Enthesitis-related arthritis. JSpA, juvenile Spondyloarthritis

Between January 2008 and December 2015, 373 patients were seen in our centre for a suspicion of ERA or JSpA. We excluded 34 patients with other diagnoses and 225 followed for less than one year, because they were mainly seen for a second opinion from our National reference centre and then included in the CEMARA database but followed afterwards in their regional centre. We included 114 patients (Fig. 1). The median follow up was 2.6 years (1.0 to 7.2 years). The baseline characteristics are presented in Table 1.

**Table 1:** Baseline characteristics

	Total, *n* = 114
Sex ratio (boys/girls)	1.7
Age at 1st symptoms, median (IQR)	9.55 (2.7)
Disease duration at the 1st visit, median (IQR)	1.2^*^ (2)
HLA-B27	49 (43%)
Familial history of SPA 1^er^ degree	32 (28%)
Familial history of psoriasis 1^er^ degree	17 (15%)
Familial history of inflammatory bowel disease	12 (10%)
Axial involvement	40 (35%)
Inflammatory back pain	23 (20%)
Sacroiliitis	33 (29%)
Peripheral arthritis	44^**^ (2%)
Oligo-articular involvement	30 (56%)
Poly-articular involvement	14 (26%)
Enthesitis	39 (72%)

*CI* confidence interval, *SpA* Spondylarthritis, *IQR* interquartile range

Axial involvement was defined as 1) inflammatory low-back pain or inflammatory dorsal pain lasting for more than one month; 2) limited spine mobility, defined by a Schober index < 10 + 4 cm; 3), sacroiliac pain at examination or alternating buttock pain; or 4) presence of axial disease by imagery. Sacroiliitis was defined as 1) sacroiliac pain at examination or alternating buttock pain; or 2) presence of sacroiliitis by either MRI or standard radiography

^*^ There was a significant difference between boys and girls (1.5 years vs. 2.3 years respectively, *p* = 0.04)

^**^ Peripheral arthritis was more frequent in boys than girls (87 vs. 67% respectively, *p* = 0.03)

At the beginning of follow-up, only 53 patients (46%) of our population were classified in the ERA group, according to the Edmonton classification criteria, with a significant difference between boys and girls (57% versus 29%, respectively, *p* <  0.01) and two patients (1.75%) in the PsA. According to the 2011 ASAS criteria, 83,patients (73%) could be classified with peripheral SpA at the first consultation, with no difference between genders (Table 2).

**Table 2:** Comparison between ASAS criteria for peripheral spondyloarthritis and current revised ILAR classification criteria for ERA

	ASAS criteria for peripheral SpA	ILAR criteria for ERA or PsA	*p*
1st consultation (%)			
All patients	83 (73%)	55 (48%)	< 0.01
Boys	55 (76%)	43 (60%)	0.03
Girls	28 (67%)	12 (29%)	< 0.01
Last follow up (%)			
All patients	105 (92%)	85 (75%)	< 0.01
Boys	65 (90%)	56 (78%)	0.04
Girls	40 (95%)	32 (76%)	0.01

*ASAS* Assessment of Spondyloarthritis International Society, *ILAR* International League of Association for Rheumatology, *ERA* enthesitis related arthritis

In our cohort, the ASAS criteria more efficiently diagnosed juvenile spondyloarthritis than the ILAR criteria. Furthermore, the ILAR criteria performed more poorly in the diagnosis of the disease during the first year in girls than boys (*p* = 0.001)

### Cumulative symptoms at the last follow up

During follow-up, 99 patients (87%) developed peripheral arthritis (Table 3), predominantly of the lower limbs (knee: 58%; hips: 46%, ankle: 38%). Table 3 presents the frequency of involvement for each joint. Enthesitis was recorded in 98 medical reports (86%) mostly in the lower limbs (Table 3): Achilles tendon or plantar fascia attachment to the calcaneus for 84 patients (74%), quadricipital tendon attachment to the knee for 53 patients (46%), and plantar fascia attachment to the metatarsal head for 45 patients (39%).

**Table 3:** Cumulative symptoms from the first to last follow up

	Total, *n* = 114
Peripheral arthritis	99 (87%)
Oligo-articular involvement	66 (58%)
Hips	52 (46%)
Knee	66 (58%)
Ankle	43 (38%)
Mild-foot	10 (9%)
Metatarsophalangeal	19 (17%)
Shoulder	14 (12%)
Elbow	14 (12%)
Wrists	28 (25%)
Metacarpo-phalangian	15 (13%)
Proximal Interphalangial	14 (12%)
Dactylitis	15^*^ (13%)
Enthesis	98 (86%)
Pelvic and greater trochanter enthesitis	25 (22%)
Knee	53 (46%)
Plantar fascia insertion into the metatarsal head	45 (39%)
Achilles’ tendon and fascia plantar insertion into the calcaneus	84 (74%)
Axial involvement	72 (63%)
Dorsal spine	27 (24%)
Lumbar spine	50 (44%)
Sacroiliac joints	54 (47%)

We followed our cohort for a median duration of 2.6 years (IQR = 2.3) and the median disease duration was 4.3 years (IQR = 3.2)

^*^ Dactylitis was more frequent in girls than boys (26 vs. 6%, respectively, *p* < 0.01)

**Fig. 2:**
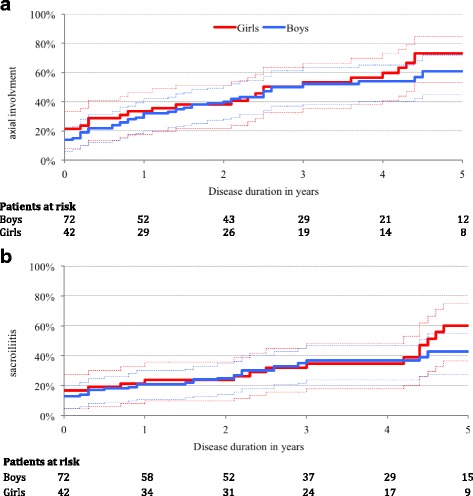
Evolution of axial disease prevalence. **a** Axial involvement (defined as: 1) inflammatory low back pain or inflammatory dorsal pain lasting for more than one month; 2) limited spine mobility; 3) sacroiliac pain at examination or intermittent buttock pain; or 4) presence of axial disease by imagery.). **b** Sacroiliitis Axial involvement was rare in the first years of the disease with a progressive linear increase in its prevalence up to 60 to 70% after five years

Axial disease was active at the latest follow-up in 72 patients (63%). Fifty-four patients (47%) developed sacroiliitis (Table 3). The median disease duration before the onset of axial symptoms or sacroiliitis was 2.6 years (IC_95_ = [2.2–4.4], Fig. 2a) and 5.3 years (IC_95_ = [4.1–7.7], Fig. 2b), respectively. After five years of evolution, Kaplan-Meier estimates showed that axial disease and sacroiliitis would be present in 66% (IC_95_ = [54.1–74.6]; Fig. 2) and 48.4% of cases (IC_95_ = [44.6–58.2]), respectively. Inflammatory back pain showed the same pattern (Additional file 2: Figure S1). There was no difference between boys and girls (*p* = 0.98).

Thirty-seven of 73 patients with inflammatory back pain (IBP) or sacroiliac tenderness (51%) underwent MRI examination. Axial involvement was confirmed for 18 patients (13/24 boys and 5/13 girls). There were 28 sacroiliac MRI for 21 patients available for review by our expert radiologist. For nine patients, the MRI were not copied on our imaging server and no CD was available for review. Our radiologist disagreed with the initial MRI interpretation for five cases (sacroiliitis had been undiagnosed in one case and overstated in four). The interrater reliability was fair as evaluated by the kappa statistic (k = 0.49, IC_95_ = [0.12–0.86]). Sacroiliitis was confirmed for five patients, based on acute lesions in all five and chronic lesions in two. Acute lesions consisted of synovitis and articular effusion in five cases and bone oedema in four. Chronic lesions consisted of erosion and sclerosis in two cases and degenerative fatty infiltration in one.

At the last follow-up, after a median disease duration of 4.3 years (1.3 to 11.2), 105 patients (92%) met the 2011 ASAS criteria for peripheral SpA, with no difference between genders. Seventy-nine patients (69%) met the Edmonton criteria for ERA and six (5.3%) those for PsA, together identifying 74.3% of the patients as having SpA, witch is significantly less than the ASAS criteria for peripheral SpA.

### Response to treatment and tolerance

Clinical improvement and clinical remission were recorded for 96 (84.2%) and 52 patients (45.6%), respectively, treated solely with NSAIDs. Polyarthritis during the first years of the disease or axial involvement were predictive of non-response to NSAID therapy (OR = 0.31, *p* = 0.06 and OR = 0.24, *p* = 0.05, respectively). The most frequent adverse effect of NSAIDs was gastralgia in 35% of cases. One serious adverse event was documented (transient acute kidney injury during viral gastroenteritis).

Anti-TNF therapy was given to 48 patients (42.1%) after a median disease duration of 5.4 years (IC_95_ = [4.6–8.0], Additional file 3: Figure S2A) Thirty-three patients (69%) presented axial disease before instauration of the first biotherapy, with a median axial disease duration of 0.6 years. All patients received NSAIDs before the introduction of anti-TNF. A second anti-TNF agent was administered to 13 patients. Anti-TNF treatment resulted in inactive disease for 58.3% of cases after six months (IC_95_ = [42.7–69.6]) and 68.6% after one year (IC_95_ = [52.0–79.5], Additional file 3: Figure S2B), with no differences between etanercept and adalimumab (*p* > 0.5). Boys had a better response than girls to both anti-TNF therapies (OR = 6.94, *p* <  0.01). Treatment tolerance was good. The most frequent adverse events were allergic reactions (five patients). One patient developed an inflammatory bowel disease after the initiation of etanercept. Another had to stop treatment because of recurrent infection.

At the latest follow-up, 63 patients (55%) were in clinical remission, including 23 (20%) off therapy.

### Prognostic factors

The only prognostic factor associated in univariate analysis with both active disease at the last follow-up and sacroiliitis was a familial history of SpA (OR = 3.1, *p* = 0.01 and OR = 3.6, p <  0.01, respectively; Table 4). This result was confirmed in multivariable analysis (OR = 3.6, *p* <  0.01 and OR = 3.1, *p* = 0.01, respectively). Enthesitis was associated with sacroiliitis (OR = 4.2, *p* = 0.04 in multivariable analysis). Male gender was the only prognostic factor associated with a lower risk of active disease at last follow up (OR = 0.3, *p* = 0.01) in univariate, but not multivariable analyses (OR = 0.4, *p* = 0.06). We did not find any other prognostic factor (Table 4).

**Table 4:** Risk factors for sacroiliitis and active disease

	Sacroiliitis		Active disease at the last follow up	
	Odds Ratio (CI 95%)	*p*	Odds Ratio (CI 95%)	*p*
	Univariate analysis			
Boys	0.7 (0.3–1.6)	0.41	0.3 (0.1–0.7)	0.01
HLA-B27	0.8 (0.3–1.9)	0.67	0.9 (0.4–2.1)	0.84
Age > 12 years	1.7 (0.6–4.9)	0.31	0.4 (0.1–1.1)	0.07
Familial history of SpA	3.6 (1.6–8.2)	0.02	3.1 (1.4–7.1)	0.01
Oligo-articular involvement at first consultation	0.5 (0.2–1.0)	0.06	0.5 (0.2–1.0)	0.06
Hip Arthritis	1.2 (0.6–2.5)	0.66	1.4 (0.7–2.9)	0.39
Enthesitis	4.7 (1.3–17.5)	0.01	1.7 (0.6–4.5)	0.32
Extra-articular involvement	1.2 (0.5–2.6)	0.69	1 (0.5–2.2)	1
	Multivariable analysis			
Boys	0.94 (0.4–2.3)	0.82	0.44 (0.2–1.1)	0.06
Age > 12 years	2.61 (08–8.8)	0.09	0.48 (0.1–1.6)	0.22
Familial history of SpA	3.61 (1.5–8.7)	< 0.01	2.98 (1.2–7.3)	0,02
Enthesitis	4.2 (1.1–16.6)	0.04	1.55 (0.5–4.9)	0.45
Oligo-articular involvement at first consultation	0.47 (0.2–1.2)	0.10	0.73 (0.3–1.7)	0.46

*CI* confidence interval

We studied the five factors (male gender, age > 12 years at disease onset, familial history of SpA, presence of enthesitis, and oligo-articular involvement at first consultation) associated with at least one of our judgment criteria for the multivariable analysis

## Discussion

This study describes the initial clinical presentation and natural history of a large cohort of patients diagnosed with JSpA/ERA and followed in a tertiary care paediatric rheumatology centre. There was progressive occurrence of axial disease in two-thirds of cases. Initial treatment consisted of NSAIDs and resulted in inactive disease in 45.6% of cases. Half of the patients received anti-TNF therapy several years after disease onset and two-thirds achieved inactive disease within one year. A family history of SpA was the main risk factor for axial disease, sacroiliitis and persistently active disease at the last follow-up. This study is based on one of the largest paediatric SpA cohorts yet published.

There was an association of male gender (Sex ratio = 1.7) and HLA-B27 (56%) with JSpA, in accordance with the literature [3, 13, 14]. The median age at disease onset (9.5 years) was similar to that reported in the literature [6, 15–19].

Our study confirmed a low rate of symptomatic axial disease at disease onset, with a marked increase, however, within the first years of the disease: 60% of our patients showed axial involvement after five years. This was also true when studying only sacroiliitis or inflammatory lumbar pain. Axial disease has generally been considered to be rare in JSpA since it and sero-negative forms were first described [20–22]. In contrast to the adult form, in which inflammatory back pain is often a major symptom at disease onset, axial involvement progressively appears months to years after the onset of JSpA [3, 22–24]. Our study showed such progressive apparition in a high proportion of patients, consistent with the recent review by R. Burgos Vargas on adult onset undifferentiated SpA and JSpA [23]. A similar progressive increase of the incidence of defined ankylosing SpA has been reported in cohorts of adult undifferentiated SpA and JSpA, with the proportion of affected patients ranging from 12% to 42% after three to five years and 19% to 90% after nine to eleven years [23].

MRI results were available for 37 patients in our study and confirmed axial disease in half, calling into question the sensitivity and specificity of clinical examination to determine axial involvement. In one study, the sensitivity of physical examination was 23% and the specificity 67.9% [25]. The lack of specificity of clinical assessment of axial symptoms is probably explained by the difficult differential diagnosis with other painful conditions, such as fibromyalgia, which may coexist in adults with SpA [26, 27]. To our knowledge, there has been no studies on the frequency of association between chronic pain syndromes, such as fibromyalgia and any type of JIA. Although MRI is a valuable tool to support axial involvement, its interpretation by non-experienced radiologists may be misleading in some cases, particularly in children, as suggested here by the only fair rate of agreement between the first radiologist and our expert radiologist who reviewed images and the low agreement between radiologists in describing acute sacroiliac lesions. Interpretation of sacroiliac MRI is challenging in children and adolescents due to the presence of thick cartilaginous growth plates which may be falsely interpreted as sacroiliitis [28]. Contrast injection may be particularly useful in such cases to show evidence, or not, of inflammation, unlike in adults in which it provides no added value over STIR sequences in MRI of the sacroiliac joints in the early detection of SpA [28].

The high response rates to NSAID therapy was consistent with that reported for adult SpA. Anti-TNF, mainly etanercept and adalimumab, were the only biological drugs used in our cohort. Most patients treated with TNF antagonists responded rapidly, resulting in inactive disease within 6 to 12 months, as also observed in retrospective studies [25] and clinical trials of etanercept [29, 30] and adalimumab for ERA or juvenile ankylosing SpA [31, 32]. Etanercept fulfilled the PedACR 70 response criteria for more than 55% of cases in the BIKER prospective cohort [33]. The proportion of our patients who achieved clinical remission was consistent with those reported in the literature, with long-term follow-up studies in adults showing remission rates between 17 and 44% [6].

Among several risk factors reported for adult SpA, a familial history of SpA was the only risk factor associated with both axial disease and sacroiliitis during follow-up, whereas the presence of enthesitis was a risk factor for sacroiliitis. Oligo-articular disease at first consultation was a negative predictor of axial disease and sacroiliitis. Published studies on JSpA contain discrepancies regarding the risks due to male gender, age, family history, hip arthritis, or articular counts [15, 34, 35]. One of the major difficulties to summarize these data is the absence of a consensus concerning the way to diagnose axial disease, some using clinical criteria, others conventional imaging techniques or MRI.

Here both univariate and multivariable analyses also showed a familial history of SpA to be the sole risk factor of active disease at the last follow-up after a median disease duration of 4.3 years. Only one study previously analysed risk factors associated with failure to achieve clinical remission: in 2006, B. Flatø et al. showed that female gender, a family history of ankylosis SpA in a first degree-relative, and ankle arthritis within the first six months were associated with failure to achieve remission after 15 years of follow-up [6].

Our study also assessed the 2011 ASAS criteria for peripheral SpA in a paediatric population, showing them to be much more sensitive than the Edmonton classification criteria for ERA, even when associated with the psoriasis JIA criteria. This may be due to several factors. First, the principle of the Edmonton criteria is that all categories are mutually exclusive. Patients who cannot be classified in any of the six types of JIA (oligo arthritis, polyarthritis rheumatoid factor negative, polyarthritis rheumatoid factor positive, ERA, PsA, or systemic arthritis) are classified as having undefined JIA. In the case of SpA, the fact that PsA is separated from other types of SpA classified in the ERA group is problematic. As discussed in the literature [36], some patients with a typical clinical picture of SpA, but with a familial history of psoriasis, are excluded from the ERA group and are classified as having undefined arthritis. On the other hand, male patients with HLA-B27 and older than six years at first symptoms are excluded from the PsA group in the Edmonton criteria. The place of psoriasis should be reconsidered in future classifications [37]. Second, ERA criteria had a lower sensitivity in girls than boys in our study, possibly may due to the fact that one ERA criterion is male gender and age over six at disease onset, leading girls with peripheral arthritis in adolescence to be mainly classified as undifferentiated JIA. The good sensitivity of ASAS criteria for peripheral SpA in our cohort, as in a previous study [38], supports the view by R. Burgos Vargas [23] that the ASAS criteria may be of particular interest in the paediatric population at disease onset, when axial involvement is usually absent.

Our study has several limitations. First, our data are retrospective, and although collected from a register, not all data were available for certain time points. Second, we used clinicians’ opinions as a gold standard to diagnose the disease and evaluate its activity because of the lack of good clinical criteria. Third, disease activity and treatment response were evaluated based on the opinion of the treating physician and the Bath Ankylosing Spondylitis Disease Activity Index (BASDAI), Juvenile Arthritis Disease Activity Score (JADAS), or other parameters of disease activity status were not systematically included. Fourth, the duration of follow-up was heterogeneous, ranging from 1.0 to 7.2 years. Fifth, only one third of patients underwent MRI. MRI examinations in our centre were performed based on the clinical judgement of the treating physician, mostly when lumbosacral pain or limited spinal mobility were present. As discussed earlier, axial disease can be clinically silent. There is no consensus on how to consider axial disease seen only by MRI with no clinical symptoms. Sixth, our population was followed in a tertiary centre, with recruitment likely biased towards more severe cases.

## Conclusion

We confirm that JSpA is characterised by its peripheral pattern at disease onset, with peripheral arthritis and enthesitis. However, axial disease developed over the next five years in more than half our patients. MRI is clearly a valuable tool to assess axial involvement, but its interpretation may be more difficult in children and require, in many cases, an experienced radiologist. Familial history of SpA is one of the major prognostic factors associated with both sacroiliitis and persistence of active disease over years, favouring the close follow-up of such patients to initiate effective and timely treatment, knowing that TNF inhibitors have proven efficacy in JSpA/ERA patients with an inadequate response to NSAIDs. The current revised Edmonton classification criteria lacked both specificity and sensitivity to classify JSpA/ERA, which merits, among other considerations, the on-going effort to improve classification criteria of JIA. Applying the 2011 ASAS criteria for peripheral SpA may be worth considering for JSpA, as they showed high sensitivity to diagnose our patients both at their first assessment and at the last follow-up.

## Additional files

Additional file 1: **Table S1.** Edmonton criteria for ERA and PsA. (DOCX 13 kb)

Additional file 2: **Figure S1.** Evolution of the prevalence of inflammatory back pain. Inflammatory back pain was defined as proposed by the ASAS in 2009 [39]. Insidious onset. Improvement with exercise. No improvement with rest. Pain at night. (DOCX 63 kb)

Additional file 3: **Figure S2.** Biological therapy: time to introduction and remission rate. Disease duration before introduction of a first line of biological therapy (years). Remission rate after introduction of a biological therapy (years). Remission was defined as the absence of any articular involvement (both peripheral and axial) and any enthesial involvement for at least six months. (DOCX 104 kb)

## Abbreviations

ASAS: Assessment of Spondyloarthritis International Society
ERA: Enthesitis-related arthritis
IBD: Inflammatory bowel disease
IQR: Inter-quartile range
JIA: Juvenile idiopathic arthritis
JSpA: Juvenile spondyloarthritis
MRI: Magnetic resonance imagery
NSAID: Non-steroidal anti-inflammatory drug
OR: Odds ratio
PsA: Psoriatic arthritis
SpA: Spondyloarthritis
TNF: Tumour necrosis factor
