# Supplementary material for: Clinical features of children with enthesitis-related juvenile idiopathic arthritis / juvenile spondyloarthritis followed in a French tertiary care pediatric rheumatology centre
Source: Pediatr Rheumatol Online J. 2018 Apr 2;16:21. doi: 10.1186/s12969-018-0238-9 (PMC5879929; doi:10.1186/s12969-018-0238-9)
Supplement: Supplementary file 1 — Table S1. Edmonton criteria for ERA and PsA. (DOCX 13 kb) [file 12969_2018_238_MOESM1_ESM.docx]

| **Table S1.:Edmonton criteria for ERA and PsA:** |
| --- |
| **Enthesitis-Related Arthritis**  Definition: Arthritis and enthesitis, or arthritis or enthesitis with at least two of the following:  1. The presence of or a history of sacroiliac joint tenderness and/or inflammatory lumbosacral pain  2. The presence of HLA-B27 antigen  3. Onset of arthritis in a male over 6 years of age  4. Acute (symptomatic) anterior uveitis  5. History of ankylosing spondylitis, enthesitis-related arthritis, sacroiliitis with inflammatory bowel disease, Reiter’s syndrome, or acute anterior uveitis in a first-degree relative  Exclusions: a, d, e. |
| **Psoriatic Arthritis**  Definition: Arthritis and psoriasis, or arthritis and at least two of the following:  1. Dactylitis  2. Nail pitting or onycholysis  3. Psoriasis in a first-degree relative  Exclusions: b, c, d, e. |
| **Exclusions**  a. Psoriasis or a history of psoriasis in the patient or first-degree relative.  b. Arthritis in an HLA-B27-positive male beginning after the 6th birthday.  c. Ankylosing spondylitis, enthesitis-related arthritis, sacroiliitis with inflammatory bowel disease, Reiter’s syndrome, or acute anterior uveitis, or a history of one of these disorders in a first-degree relative.  d. The presence of IgM rheumatoid factor on at least two occasions at least three months apart.  e. The presence of systemic JIA in the patient. |
