# Supplementary material for: Clinical features of children with enthesitis-related juvenile idiopathic arthritis / juvenile spondyloarthritis followed in a French tertiary care pediatric rheumatology centre
Source: Pediatr Rheumatol Online J. 2018 Apr 2;16:21. doi: 10.1186/s12969-018-0238-9 (PMC5879929; doi:10.1186/s12969-018-0238-9)
Supplement: Supplementary file 2 — Figure S1. Evolution of the prevalence of inflammatory back pain. Inflammatory back pain was defined as proposed by the ASAS in 2009 [39]. Insidious onset. Improvement with exercise. No improvement with rest. Pain at night. (DOCX 63 kb) [file 12969_2018_238_MOESM2_ESM.docx]

|  |
| --- |
| \| **Patients at risk** \| \| \| \| \| \| \| \| \| --- \| --- \| --- \| --- \| --- \| --- \| --- \| --- \| \| Boys \| 72 \| 63 \| 53 \|  \| 38 \| 26 \| 15 \| \| Girls \| 42 \| 37 \| 32 \|  \| 26 \| 19 \| 15 \| |
