# Supplementary material for: Clinical features of children with enthesitis-related juvenile idiopathic arthritis / juvenile spondyloarthritis followed in a French tertiary care pediatric rheumatology centre
Source: Pediatr Rheumatol Online J. 2018 Apr 2;16:21. doi: 10.1186/s12969-018-0238-9 (PMC5879929; doi:10.1186/s12969-018-0238-9)
Supplement: Supplementary file 3 — Figure S2. Biological therapy: time to introduction and remission rate. Disease duration before introduction of a first line of biological therapy (years). Remission rate after introduction of a biological therapy (years). Remission was defined as the absence of any articular involvement (both peripheral and axial) and any enthesial involvement for at least six months. (DOCX 104 kb) [file 12969_2018_238_MOESM3_ESM.docx]

|  |
| --- |
| \| **Patients at risk** \| \| \| \| \| \| --- \| --- \| --- \| --- \| --- \| \|  \| 113 \| 108 \| 89 \| 69 \| \| 43 \| 28 \| |
|  |
| \| **Patients at risk** \| \| \| \| \| \| \| --- \| --- \| --- \| --- \| --- \| --- \| \| Eta \| 39 \| 14 \| 7 \| 6 \| 2 \| - \| \| Ada \| 22 \| 10 \| 7 \| 5 \| 4 \| - \| |

**a**
